# Formation of buried superconducting Mo_2_N by nitrogen-ion-implantation†

**DOI:** 10.1039/d0ra08533b

**Published:** 2020-12-16

**Authors:** Joonhyuk Lee, Jun Kue Park, Joon Woo Lee, Yunseok Heo, Yoon Seok Oh, Jae S. Lee, Jinhyung Cho, Hyoungjeen Jeen

**Affiliations:** Department of Physics, Pusan National University Busan 46241 Korea hjeen@pusan.ac.kr; Korea Multi-purpose Accelerator Complex, Korea Atomic Energy Research Institute Gyeongju 38180 Korea; Department of Physics, Ulsan National Institute of Science and Technology Ulsan 44919 Korea; Department of Physics Education, Pusan National University Busan 46241 Korea

## Abstract

Nitrogen ion implantation is a useful technique to put nitrogen ions into lattices. In this work, nitrogen ion implantation into epitaxial Mo films is performed to create a buried superconducting γ-Mo_2_N. Atomically flat epitaxial (110) Mo films are grown on (0001) Al_2_O_3_. By impinging nitrogen ions, where the beam energy is fixed to 20 keV, we observe (111) γ-Mo_2_N diffraction and the formation of a γ-Mo_2_N layer from X-ray reflectivity. Magnetization and transport measurements clearly support a superconducting layer in the implanted film. Our strategy shows that formation of a buried superconducting layer can be achieved through ion implantation and self-annealing.

## Abstract 2

Formation of chemically distinct interfaces, including crystalline buried-superconducting Mo_2_N, by low-energy nitrogen ion implantation in an epitaxial molybdenum thin film.

## Graphical abstract

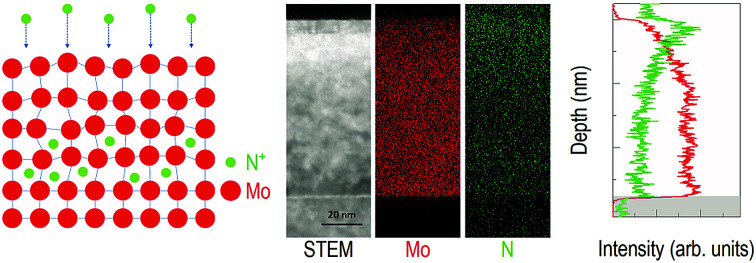

## Introduction

Ion implantation is a versatile technique to incorporate ions into crystalline lattices.^1,2^ Through ion implantation, electrical, magnetic, and optical properties have been tuned. Fractional boron doping into silicon using ion implantation enabled formation of the desired level of doping in semiconductors.^3,4^ In addition, for fabricating dilute magnetic semiconductors, magnetic ion implantation was used for room temperature ferromagnetism.^2,5–7^ Unusual luminescence and photo-activity in the implanted films were also reported.^8–11^ However, the implantation strategy is not limited to semiconductors. In recent, it was adopted for stabilizing meta-stable phase such as rare-earth-free permanent magnet such as Fe_16_N_2_ by implanting nitrogen ions into iron lattices.^12^ Thus, combining ion implantation technique in crystal synthesis may bring an another degree of freedom for tuning materials properties. In this regard, creation of a metal nitride from nitrogen ion implantation is important, since nitrogen molecules are normally very stable. So, it is not easy to decompose nitrogen molecules, incorporate nitrogen ions into the lattices, and form desired stoichiometry. Ones often used ammonia as a processing agent for nitridation.^13–15^ Even if ion implantation is rather destructive method to impinge small atomic or molecular nitrogen ions, usually N^+^ or N_2_^+^, into crystal lattices, it is expected to intercalate nitrogen ions effectively and stabilize them in the lattice through post-process: *i.e.* heat treatment. This leads to stabilize a highly-stabilized resistive surface and/or exotic physical properties like superconductivity, which will be introduced in this work.

Nitridation of molybdenum using ion implantation is of considerable interest, since molybdenum nitrides can be mechanically strong and superconducting materials with different critical temperatures depending on nitrogen content.^16–21^ It has been proven that ion beam implantation with wider nitrogen beam energy (up to 200 keV) and relatively high nitrogen ion dose (10^16^ ∼ 10^17^ ions cm^−2^) can induce the formation of γ-Mo_2_N, δ-MoN, and B_1_–MoN. It is generally known that higher ion incorporation could be possible, when lower energy and higher dose used.^20^ In this work, we observed evidence of buried superconducting-phase formation by ion implantation on (110) epitaxial Mo thin films using relatively low energy. First, we synthesized atomically flat (110) Mo thin films. The films were transferred to ion beam facility for atomic nitrogen ion (N^+^) beam implantation at low energy to minimize disordering of Mo atoms. The implanted films were tested to find potential formation of superconducting nitrides using X-ray scattering, cross-section transmission electron microscopy, atomic force microscopy (AFM), transport, and magnetization measurements.

## Experimental

**Fig. 1:**
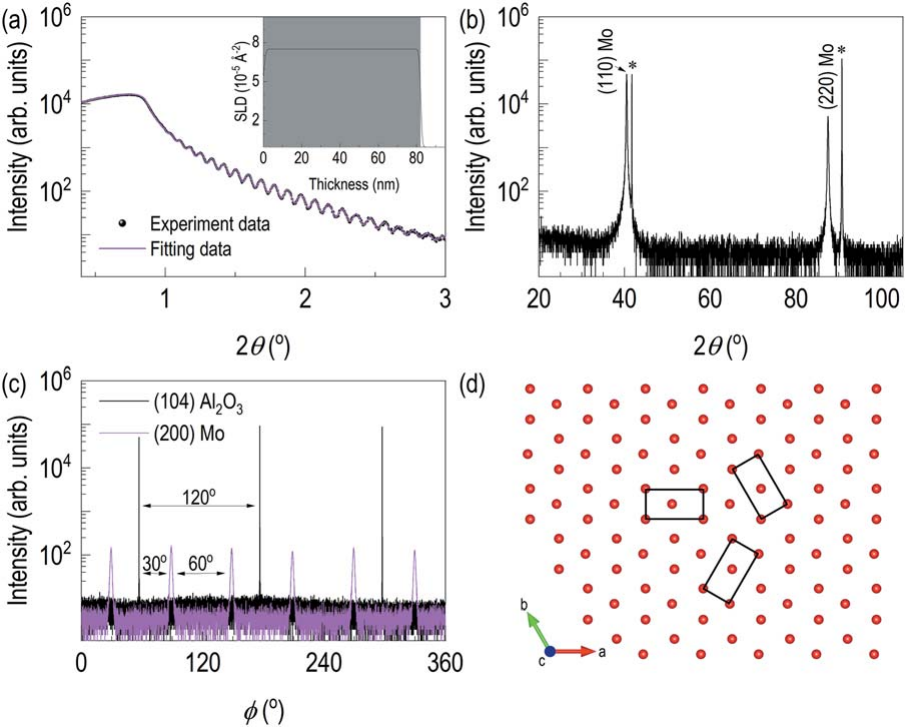
(a) X-ray reflectivity and its fitting of the epitaxial Mo thin film on (0001) Al_2_O_3_. Inset shows depth profile of electron scattering length density. (b) X-ray diffraction patterns of (110) Mo thin film. (c) *φ* scans of (104) Al_2_O_3_ and (200) Mo. (d) Based on *φ* scan results, it expected that textured Mo and associated epitaxy relation is proposed.

80 nm-thick epitaxial Mo thin films were grown on (0001) Al_2_O_3_ substrates (Crystal bank, Pusan National University) using custom-made DC magnetron sputtering. The detailed growth condition for Mo films are following: 5 mTorr as forming gas pressure (*P*_forming_), 50 W of DC power, and 700 °C of substrate temperature (*T*_S_). X-ray diffraction (D8 Discover, Bruker) techniques such as X-ray reflectivity, 2*θ*–*ω* scan, *φ*-scan were employed to characterize structural information of epitaxial Mo films (see Fig. 1). After confirming epitaxial growth of Mo films, N^+^ beam implantation experiments were performed in Korea Multi-purpose Accelerator Complex. Beam condition was fixed at 20 keV with different ion doses: 10^15^, 10^16^, and 5 × 10^16^ ions cm^−2^. To predict potential ion distribution, we used the transport of ions in matter (TRIM) results^22^ to estimate profile of implanted nitrogen ions and recoiled or disordered molybdenum ions. X-ray 2*θ*–*ω* scan and X-ray reflectometry (XRR) were specially adopted to see formation of buried molybdenum nitrides and destabilization of molybdenum by ion implantation. To observe microstructure and chemical inhomogeneity due to nitrogen ion implantation, cross-sectional transmission microscope (TALOS F200X, FEI) was used. Z-contrast imaging and energy dispersive X-ray spectroscopy (EDS) are performed on the highest nitrogen dosed sample (5 × 10^16^ ions cm^−2^ of N^+^). X-ray reflectivity fitting was performed with GenX software.^22^ After confirming the formation of the nitrides, we used SQUID magnetometer (MPMS3, Quantum Design) and Physical Property Measurement System (Quantum design) to observe temperature dependence of magnetization and transport property. For a SQUID measurement, we used 100 Oe of magnetic field. For a transport measurement, we used conventional four probe transport geometry and the data is normalized by resistance value at *T* = 8 K (normal state).

## Results and discussion

Fig. 1 showed the evidence of epitaxial synthesis of Mo films. Fig. 1(a) showed X-ray reflectivity of Mo films on (0001) Al_2_O_3_. Clear Kiessig fringes show well-defined interfaces with about 80 nm in thickness. The obtained electron scattering length density (eSLD) of a Mo layer is 7.54 Å^−2^, which is very similar to the bulk value of Mo (eSLD = 7.64 Å^−2^). In addition, from X-ray reflectivity fitting, we also obtained the surface roughness information, which is about 1 nm. The value is similar to the value obtained from atomic force microscopy (see Fig. S1†) Fig. 1(b) showed 2*θ*–*ω* scan. It showed (110) Mo is stabilized on (0001) Al_2_O_3_. In addition to the determination of out-of-plane information, we performed off-axis *φ* scan to figure out complete epitaxial relationship. *φ* scans around (104) Al_2_O_3_ and (200) Mo were selected. We observed Al_2_O_3_ peaks with 120° apart, while we obtained six peaks from (200) Mo with 60° apart. Also, Mo peaks are 30° apart from the nearest Al_2_O_3_ peaks. It indicates potential texture in the plane. In Fig. 1(d), lattice oxygens on (0001) Al_2_O_3_ are shown with rectangular (110) Mo lattices. From the figure, it can be easily seen that [001] Mo does not coincide with [100] Al_2_O_3_. In addition, [001] Mo is at least 30° apart from [100] Al_2_O_3_.

**Fig. 2:**
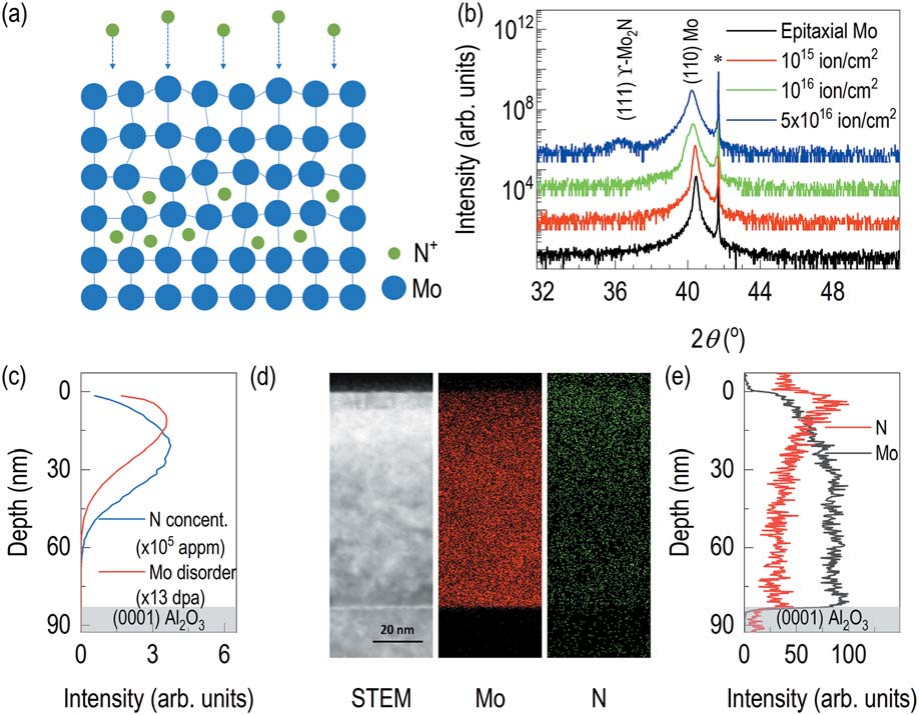
(a) Schematic diagram in case of nitrogen implantation, (b) XRD results on different doses with same beam energy, (c) simulated distribution of nitrogen and recoiled molybdenum along depth, (d) cross-sectional transmission electron microscopy results: Z-contrast image, EDS of Mo, and EDS of N, and (e) depth profile of nitrogen and molybdenum as a function of depth from EDS.

After confirming epitaxial synthesis of (110) Mo thin film on (0001) Al_2_O_3_, we performed nitrogen-ion implantation with 20 keV and various doses. A schematic diagram in Fig. 2(a) describes how nitrogen ions may be intercalated. Since ion energy is high enough, it creates recoiling of Mo ions from its equilibrium positions. Fig. 2(b) shows XRD results of Mo films with various doses of the implantation. First, there is no shoulder peak near the substrate peak, which is likely to be associated with the effect of lattice distortion or implanted ions. It is surprising that a new diffraction peak was observed in addition to the broadening of (110) Mo peak. (110) Mo peak are broadened and shifted toward lower 2*θ* angle as the dose increases. It indicates, by recoiling of Mo atoms, lattice expansion is taken place. Note that the significant lattice expansion was found, when the dose is above 10^16^ ions cm^−2^. In addition, when we checked rocking curve of (110) Mo, we observed its full width half maximum (FWHM) changes from 0.08° for as-grown Mo film to 0.14° for N^+^ implanted Mo film with 5 × 10^16^ ions cm^−2^. A new diffraction peak is shown when the dose reached 5 × 10^16^ ions cm^−2^. The new peak is located to that of (111) γ-Mo_2_N.^20,23^ The ion implantation experiments were performed by cooling the backplate of sample stage using chilled water, and the temperature of backplate is kept to 24 °C. It is likely that the temperature of Mo films during ion implantation is different from that of backplate potentially due to self-annealing during ion implantation,^24–26^ which will be a potential reason for formation of crystalline molybdenum nitride. From AFM results in Fig. S1,† the surface roughness of the ion-implanted films is about 2 nm, which is higher than the value from an as-grown Mo film. Interestingly, as the dose increases, the grain size increases but the surface roughness decreases. It can be evidence of self-annealing of the surface through ion beam implantation.

After finding the formation new phase in 5 × 10^16^ ions cm^−2^ of nitrogen ion dose, to estimate depth information of recoiled Mo and implanted nitrogen, TRIM was used to simulate distribution of nitrogen ions in the Mo film and distribution of recoiled Mo atoms (Fig. 2(c)). It is noted that surface Mo atoms are likely to lose their equilibrium positions during nitrogen implantation. From our TRIM simulation, the distribution of recoiled Mo atoms is limited to the surface. However, nitrogen ion distribution is bit different. Its center position is likely to locate deeper than that of recoiled Mo. In addition, the simulation shows nitrogen ions will reside within the 80 nm-thick Mo films. Thus, it is unlikely that the results of physical properties are originated from the modification of Al_2_O_3_ by nitrogen ions. Scanning transmission electron microscopy was also performed on the sample with 5 × 10^16^ ions cm^−2^. A Z-contrast imaging in Fig. 2(d) show clear contrasts. The first region is recognized from top surface down to 7 nm below the surface. Second layer is formed in between 7 nm and 20 nm from the top surface. It is likely due to changes in chemical composition and density. So, we additionally performed EDS of Mo and N. For the case of EDS Mo, it is clearly seen that less bright signals near the surface. Fig. 2(e) shows depth profile of relative Mo signals from EDS. The result shows the region up to 4 nm from the surface is low density, which is less than 50% of the signals from the bulk region, found at 32 nm and below from the surface. The depth profile of Mo signal from EDS shows 20 keV of nitrogen ion beam significantly disorders Mo layer near the surface. Also, Fig. 2(d) and (e) include information of implanted nitrogen. It is clearly seen that brighter region is found near the surface. However, at the proximity of the surface, relative nitrogen dose is not the high. It indicates potential formation of the buried Mo_2_N superconducting layer. From the depth profile of relative N signals from EDS, the highest nitrogen signal is found at 4 nm below the surface. It is seen that sufficient amount of nitrogen ions are found down to 30 nm. From STEM/EDS, it is clearly seen that nitrogen implantation in Mo layer, disordering of Mo layer, and no effect on Al_2_O_3_. Fig. S2 (b) and (c)† are FFTs from the lattice image in Fig. S2(a).† The zone axes are determined as [−1 1 −1] of Mo and [0 −1 1] of γ-Mo_2_N, which are well-matched with the simulation results. In addition, we clearly observed lattice expansion upon nitrogen implantation.

**Fig. 3:**
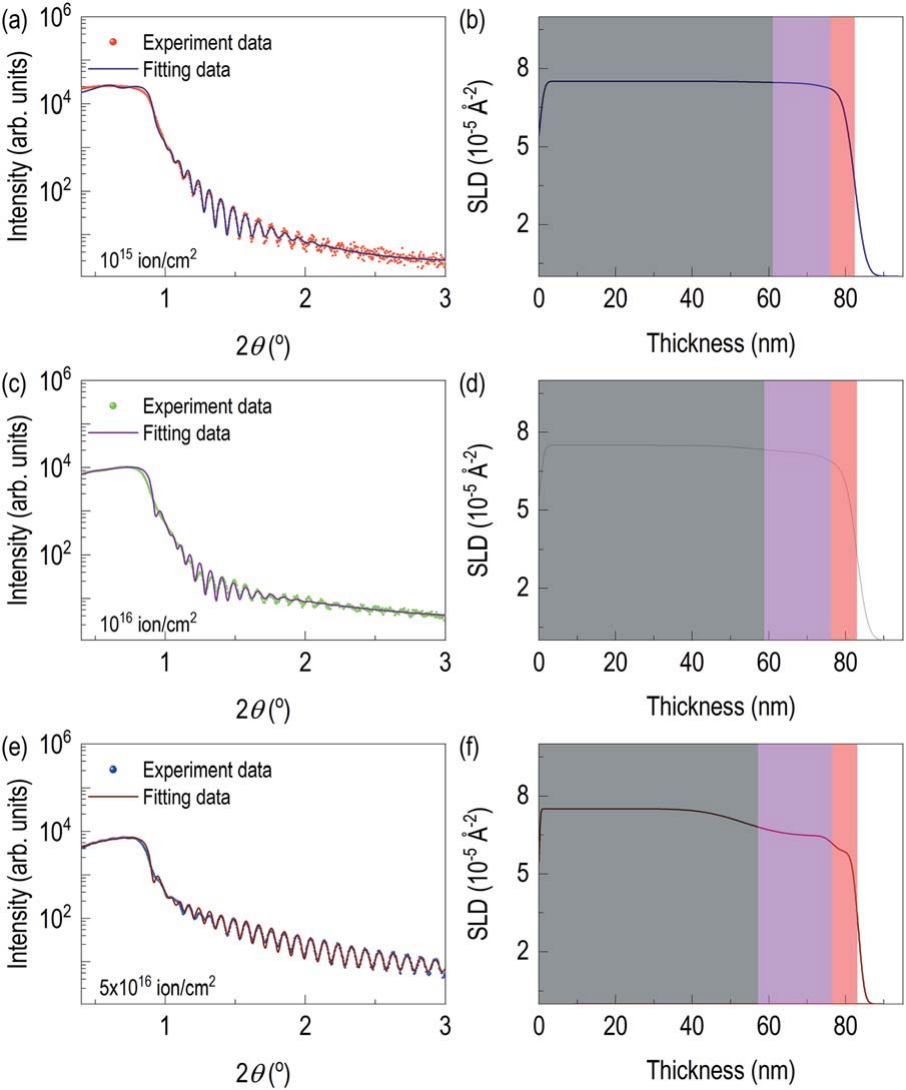
Experimental XRR curves (circle) and fitting results (solid line) of nitrogen-ion-implanted Mo film and electron scattering density from XRR fitting: (a) and (b) from 10^15^ ions cm^−2^ of N^+^ dose, (c) and (d) 10^16^ ions cm^−2^ of N^+^ dose, (e) and (f) from 5 × 10^16^ ions cm^−2^ of N^+^ dose.

After checking the formation of γ-Mo_2_N from X-ray diffraction and chemical depth profile of the highly dosed sample, we performed X-ray reflectivity of N^+^ implanted Mo films. Fig. 3 shows X-ray reflectivity, fitting, and depth profile of electron scattering length density (eSLD). In all cases, we observed clear kiessig fringes. While Fig. 3(a) is less pronounced, Fig. 3(c) and (e) show clear evidence of lattice modulation, since they show non-monotonic decay of X-ray reflectivity. In order to reflect the results of TRIM simulation and STEM/EDS results, we modeled the system with three layers: (i) defective-surface Mo layer possibly due to recoiled Mo atoms, (ii) nitrogen-implanted Mo layer, and (iii) unperturbed Mo layer. XRR fitting was performed on the XRR data from the film with 5 × 10^16^ ions cm^−2^, since it is expected to have the highest contrast due to high concentration of recoiled Mo and high dose of N^+^. After getting thickness parameters, we performed XRR fitting of other two samples, which are chemically less distinct. From XRR fitting of the film with 5 × 10^16^ ions cm^−2^, thicknesses of recoiled Mo layer, nitrogen implanted Mo layer, and unperturbed Mo layer are 6.64 nm, 19.31 nm, and 57.16 nm, respectively. The corresponding electron scattering length density (eSLD) of recoiled Mo layer, nitrogen implanted Mo layer, and unperturbed Mo layer are 5.87, 6.48, and 7.54 Å^−2^. Note that when comparing the eSLD values of the nitrogen-implanted Mo layer, the value is similar to that of γ-Mo_2_N within 3% of error.^27^

It confirms ion beam implantation creates three distinct layers (see Table 1) as we saw in Z-contrast imaging. Also, large amount of volume is still from unreacted Mo layer. Note that the eSLD of recoiled Mo layer is significantly lower value, and this may be due to continuous damage at the surface, which is related to disorder of Mo atoms. After getting full information of the highly dosed Mo films, XRR fitting of the remaining samples was performed. There are three major changes on recoiled-Mo layer and nitrogen-implanted Mo layer. Electronic SLD values of the recoiled-Mo layer are progressively decreasing with higher doses: 7.08 Å^−2^ for the case of 10^15^ ions cm^−2^, 6.60 Å^−2^ for the case of 10^16^ ions cm^−2^, and 5.87 Å^−2^ for the case of 5 × 10^16^ ions cm^−2^. It is rather drastic change above 10^16^ ions cm^−2^. However, eSLDs of nitrogen-implanted Mo layer are monotonically reduced by increase of dose: 7.45 Å^−2^ for the case of 10^15^ ions cm^−2^, 7.20 for the case of 10^16^ ions cm^−2^, and 6.48 for the case of 5 × 10^16^ ions cm^−2^. Lastly, we tracked roughness of each layer. Interestingly roughness of both nitrogen-implanted Mo layer and unperturbed Mo layer are high for cases of the lower ion dose, while for the case of 5 × 10^16^ ions cm^−2^, interfacial roughness significantly reduced. Note that the surface roughness of the ion-implanted films was significantly reduced. The results from XRR fitting is consistent with those of AFM in Fig. S1.†

**Table 1:** Thickness, eSLD, and roughness values obtained from XRR model fitting at each sample. (*t*_xrr_: thickness from XRR, *r*_xrr_: surface or interface roughness from XRR)

	*t*_xrr_ (nm)	eSLD (10^−5^ Å^−2^)	*r*_xrr_ (nm)	*t*_xrr_ (nm)	eSLD (10^−5^ Å^−2^)	*r*_xrr_ (nm)	*t*_xrr_ (nm)	eSLD (10^−5^ Å^−2^)	*r*_xrr_ (nm)	*t*_xrr_ (nm)	eSLD (10^−5^ Å^−2^)	*r*_xrr_ (nm)
Defective surface Mo layer				6.44	7.08	2.30	6.81	6.60	2.27	6.64	5.87	1.29
Nitrogen implanter Mo layer				15.10	7.45	4.24	17.37	7.20	3.16	19.31	6.48	1.65
Unperturbed Mo layer	81.07	7.54	0.72	60.90	7.54	10.32	58.81	7.54	9.97	57.16	7.54	8.28
	Epitaxial Mo			10^15^ ion cm^−2^			10^16^ ion cm^−2^			5 × 10^16^ ion cm^−2^		

**Fig. 4:**
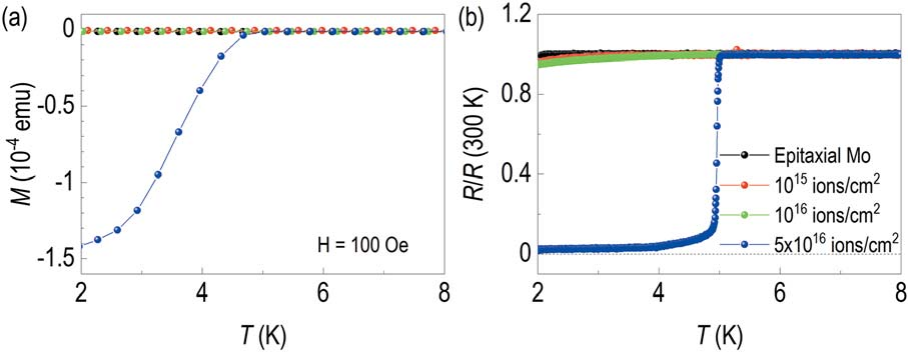
(a) Transport properties and (b) SQUID magnetization data of each sample. In both cases, superconductivity below 5 K is clearly seen from 5 × 10^16^ ion cm^−2^ of N^+^ dose.

As a buried γ-Mo_2_N layer is expected to be superconducting, we performed transport measurements and temperature dependent magnetization. First, Fig. 4(a) shows temperature dependent magnetization data. We used 100 Oe of magnetization to observe diamagnetic signal. The sample with 5 × 10^16^ ions cm^−2^ shows clear diamagnetic signal below 5 K. However, other lower-dosed samples are not diamagnetic. The superconducting critical temperatures of γ-Mo_2_N from other groups are listed in Table 2. Note that methods to make γ-Mo_2_N include solid state reaction,^16^ ion beam implantation,^28^ pulsed laser deposition,^29^ sputtering,^30–34^ ion beam assisted deposition,^34^ electron beam evaporation,^20,35^ plasma immersion ion implantation^36,37^ and post annealing^28,36,37^. However, in these papers, there was no information of superconducting critical temperatures, so it was not included in Table 2. In the table, the superconducting critical temperatures are ranged from 2.8 to 7 K, depending on growth method. We would like to emphasize bulk *T*_c_ is 5 K.^21,23,38–42^

**Table 2:** Superconducting critical temperature of γ-Mo_2_N

Mo–N Phase	Growth method	*T*_c_ (K)	ref.
γ-Mo_2_N	Solid state reaction	5	38
γ-Mo_2_N	Solid state reaction	5.5	21
γ-Mo_2_N	Powder	5.2	23
γ-Mo_2_N	Molecular beam epitaxy	2.8–3	39
Cubic-Mo_2_N	Chemical solution deposition(spin-coat)	4.5	40
Cubic-Mo_2_N	DC sputtering	6–7	41
γ-Mo_2_N	Sputtering → N^2+^ ion beam	3.8	42

This feature of the superconducting zero resistance is also clearly seen in the temperature dependence of resistance in Fig. 4(b). 20 keV beam energy of the 5 × 10^16^ ions cm^−2^ shows superconducting transition at around 5 K. It's ascribed to creation of the γ-Mo_2_N layer through ion implantation. We found small residual resistance of our superconducting sample. The process may not form a perfect defect-free superconducting layer due to the nature of ion implantation. Note that we observed characteristic slope changes from transport results of the low fluence Mo films. This indicates the films are not superconducting at the given temperature ranges, but it is possible to see some difference in superconducting critical temperature at the lower than 1.8 K. Note that *T*_c_ of pure Mo is 1 K.^43^

## Conclusions

In conclusion, we observed clear a buried superconducting layer in an epitaxial (110) Mo film grown on (0001) Al_2_O_3_ by low energy nitrogen ion implantation. The realization of superconductivity is seen with 5 × 10^16^ ions cm^−2^ and 20 keV of atomic nitrogen ion beam. It was checked that structural changes were observed through ion implantation, and the new peak was determined to be (111) γ-Mo_2_N. We performed the model fitting with three-layer model, and through eSLD and layer tracking, we could trace the γ-Mo_2_N layer formed on epitaxial Mo.

## Conflicts of interest

There are no conflicts to declare.

## Supplementary Material

RA-010-D0RA08533B-s001
