# Supplementary material for: Formation of buried superconducting Mo2N by nitrogen-ion-implantation
Source: RSC Adv. 2020 Dec 16;10(72):44339–43. doi: 10.1039/d0ra08533b (PMC9058446; doi:10.1039/d0ra08533b)
Supplement: RA-010-D0RA08533B-s001 [file RA-010-D0RA08533B-s001.pdf]

Electronic Supplementary Material (ESI) for RSC Advances.
This journal is © The Royal Society of Chemistry 2020

Supplementary Material

Formation of buried superconducting Mo_2N by nitrogen-ion-implantation

Joonhyuk Lee,[a] Jun Kue Park,[b] Joon Woo Lee,[c] Yunseok Heo,[a] Yoon Seok Oh,[c] Jae S. Lee,[b] Jinhyung Cho,[d] and Hyoungjeen Jeen*[a]

[a]Department of Physics, Pusan National University, Busan 46241, Korea.; E-mail: hjeen@pusan.ac.kr
[b]Korea Multi-Purpose Accelerator Complex, Korea Atomic Energy Research Institute, Gyeongju 38180, Korea
[c]Department of Physics, Ulsan National Institute of Science and Technology, Ulsan 44919, Korea
[d]Department of Physics Education, Pusan National University, Busan 46241, Korea

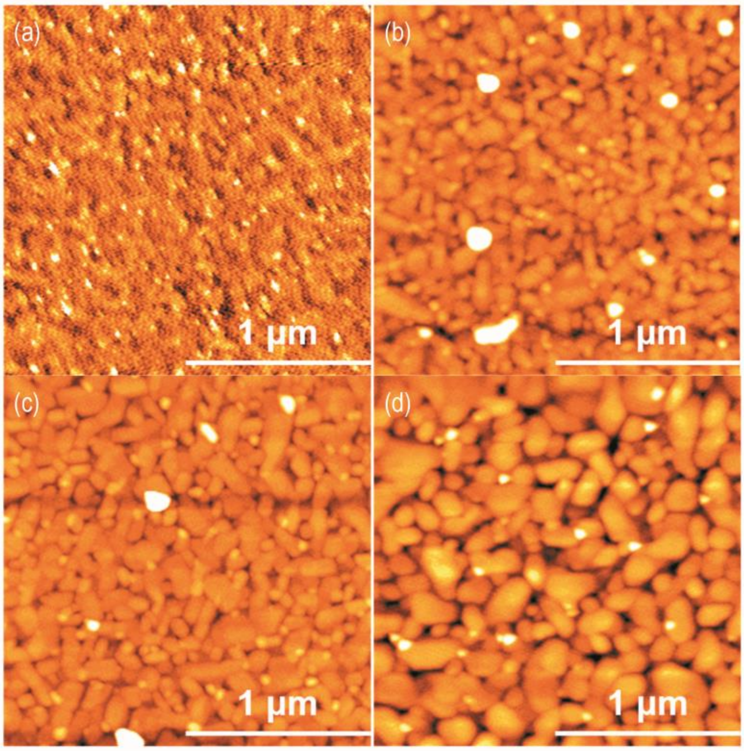

Figure S1 AFM images of (a) as-grown Mo film and nitrogen-ion implanted Mo films with different doses: (b) 10^{15} ions/cm^2, (c) 10^{16} ions/cm^2, (d) 5 x 10^{16} ions/cm^2. The r.m.s roughness values are 0.58 nm from an as-grown Mo film, 2.47 nm from the implanted film with 10^{15} ions/cm^2, 1.87 nm from the implanted film with 10^{16} ions/cm^2, and 1.66 nm from the implanted film with 5 x 10^{16} ions/cm^2. It can be interpreted as surface damage at the lower dose and the grain growth and surface smoothening due to prolonged heating driven by ion implantation.

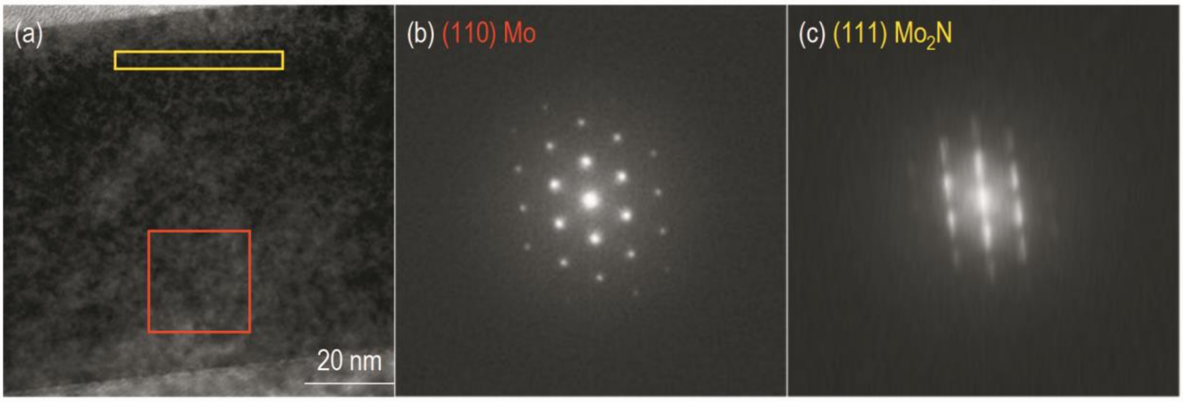

Figure S2 (a) cross-sectional TEM image of nitrogen-ion implanted Mo film with 5 x 10^{16} ions/cm^2 and fast Fourier transformation (FFT) of (b) (110) Mo film and FFT of (c) (111) γ-Mo_2N layer. The zone axes are [-1 1 -1] of Mo and [0 -1 1] of γ-Mo_2N, which are matched with the simulation software (Single Crystal, UK). Calculating the lattice constant along [110] from the FFT of (110) Mo is 4.60 nm. This is similar to the known value (4.46 nm). The lattice constant along [111] γ-Mo_2N is 7.159 nm, similar with the known value (7.208 nm).
